# Ethical issues in genomic research on the African continent: experiences and challenges to ethics review committees

**DOI:** 10.1186/s40246-014-0015-x

**Published:** 2014-08-21

**Authors:** Michèle Ramsay, Jantina de Vries, Himla Soodyall, Shane A Norris, Osman Sankoh

**Affiliations:** 1 Sydney Brenner Institute for Molecular Bioscience, University of the Witwatersrand, Johannesburg, South Africa; 2 Division of Human Genetics, National Health Laboratory Service and School of Pathology, Faculty of Health Sciences, University of the Witwatersrand, Johannesburg, South Africa; 3 Department of Medicine, UCT Centre for Clinical Research, Old Main Building, Groote Schuur Hospital, Observatory, University of Cape Town, Cape Town, South Africa; 4 MRC/Wits Developmental Pathways for Health Research Unit, Department of Paediatrics, School of Clinical Medicine, Faculty of Health Sciences, University of the Witwatersrand, Johannesburg, South Africa; 5 International Network for the Demographic Evaluation of Populations and Their Health in Low- and Middle-Income Countries (INDEPTH), Accra, Ghana; 6 School of Public Health, University of the Witwatersrand, Johannesburg, South Africa; 7 Hanoi Medical University, Hanoi, Vietnam

**Keywords:** Genomics, Ethics, Informed consent, Broad consent, Community engagement, Sample sharing, Data sharing and benefit sharing, Burkina faso, Ghana, Kenya, South Africa, Tanzania

## Abstract

This is a report on a workshop titled ‘Ethics for genomic research across five African countries: Guidelines, experiences and challenges’, University of the Witwatersrand, Johannesburg, South Africa, 10 and 11 December 2012. The workshop was hosted by the Wits-INDEPTH partnership, AWI-Gen, as part of the H3Africa Consortium.

## 

One of the primary ethical challenges to genomic research in Africa pertains to sensitivities and questions raised by communities based on their prior experiences as well as regional cultural beliefs and practices. In order for genomic research in Africa to be successful, issues like community engagement, broad consent, and the implications of sharing DNA samples and genetic data need to be debated and the potential risks of stigmatisation and harm need to be considered carefully. Ultimately, these ethical challenges need to be weighed against strategies and approaches making use of genomic research in addressing the high burden of disease in Africa.

## Background to the workshop

The Wits-INDEPTH (University of the Witwatersrand - International Network for the Demographic Evaluation of Populations and Their Health in Low- and Middle-Income Countries) Collaborative Centre, known by the abbreviation AWI-Gen (Africa Wits-INDEPTH Partnership for the Genomic Research), was established in 2012 under the auspices of the Human Health and Heredity in Africa (H3Africa) Consortium. In bringing together a multidisciplinary team of researchers, AWI-Gen is well poised to embark on a study of cardiometabolic disease risk in Africa. More specifically, the project aims to identify genetic factors that contribute to body composition, including obesity, which together with environmental factors, contribute to susceptibility for cardiometabolic diseases.

The partnership includes five health and demographic surveillance system (HDSS) field sites of the INDEPTH Network (www.indepth-network.org) across four countries, Ghana, Burkina Faso, Kenya and South Africa, and an urban study site in Soweto (Birth to Twenty and other studies within the Developmental Pathways for Health Research Unit at the University of the Witwatersrand, Johannesburg). The HDSS centres offer established infrastructure, trained fieldworkers, long-standing community engagement and detailed longitudinal phenotypic data. The key strengths of the study are harmonised phenotyping across sites, building on strong existing cohorts and representation of the geographic and social variability of African populations. This research requires a different type of involvement with the research participants, to that which has been conducted previously by INDEPTH. Since participants are required to consent to donating blood samples with broad consent and to biobanking of the biological samples, many challenges exist in communicating this information to the participants and understanding the cultural practices of the different communities among whom we plan to conduct the research.

To gain a better understanding of the ethical challenges relating to our genomic work, we invited members of ethics committees and researchers from the various regions in Africa we work in to a workshop in Johannesburg to discuss and map the ethico-legal research environment in these countries. Among the issues given priority were community engagement, informed consent, sample and data sharing, as well as benefit sharing.

Since all participating research groups have to satisfy the requirements of their institutional (and in some cases national) ethics committees in keeping with their country's policies, this workshop, hosted by AWI-Gen, was intended to stimulate discussion among African scholars and to raise the importance of growing and shaping the landscape of genomic research on the African continent in a favourable way.

## Workshop focus

The rationale for inviting members of ethics committees was to provide oversight to each of our participating institutions and also included Tanzania, as a potential future partner in our genomic research. Where appropriate, we attempted to invite individuals from institutional and national review committees. We asked participants to prepare a presentation on aspects of the ethical and legal context in their countries pertinent to genomics research. Specifically, we asked meeting invitees to consider the question: ‘How would ethics review committees in your country view genomic research and what issues would most influence their decision to approve such research?’ The purpose of these presentations was to develop a better understanding of potential bottlenecks and pertinent ethical issues in genomic research in Africa. In order to frame the discussions for the second part of the workshop, we invited an experienced ethicist (JdV) who was involved in the MalariaGen Project to highlight some of the ethical challenges they faced and how they addressed them. In the afternoon, we had breakaway sessions in which participants were asked to form small groups of five to eight to discuss one of four topics of ethical importance that we had identified. These were community engagement, broad informed consent, sample and data sharing and benefit sharing.

## Ethical oversight and perspectives

Members from the ethics review committees for each participant country gave an overview of the ethico-legal framework for research in their countries (summarised in Table 1) and provided insight into their processes and experiences. Several common concerns were raised. In particular, with reference to genomic research, these included the broad themes of participant protection, management and sharing of data and samples and appropriate recognition for the work of local scientists.

**Table 1:** Regulatory framework for ethics approval for biomedical research in five African countries

**Country**^**a**^	**Institutional and national ethics review boards**	**Process for approval of genomic research**	**Legislation**
Burkina Faso	Institutional review (only three in the country) and national review (National Ethics Committee, constituted in 2002, Ministry of Health)	Sequential manner (institutional and then national) (submitted in French)	Under auspices of the Ministry of Health
Ghana	Each health research institute has its own institutional review and institutions that fall under the Ghana Health Service also require additional review by the Ghana Health Service Ethical Review Board in Accra	Scientific approval from the institution precedes submission for ethics review	Ghana Health Service
			Data protection Act (2012)
		For international projects, an appendix with the Ghana specific protocol must be submitted	
		Sequential submission for review	
Kenya	Institutional review and nationally accredited ethics review boards	Institutional Scientific Steering Committee approval prior to submission for Institutional Ethics review. National review is done at the research institutions with accredited ethics review committees	Science and Technology Act (2001)
	(Previously Kenya Medical Research Institute (KEMRI) Ethics Review Committee approval required for biomedical research)		National Council for Science and Technology (NCST) Guidelines for Ethical Conduct of Biomedical Research Involving Human Subjects in Kenya (2004)
			Ministry of Health (MOH) - Human Biological Materials
South Africa	Universities and the medical research council have their own institutional ethics committees	Only need approval from institutional ethics committee (hospital-based research requires additional approval from the hospital and research in rural communities requires provincial approval)	National Health Act No. 61 (2003)
			National Research Ethics Council (http://www.nhrec.org.za)
			Export permit required from the Department of Health for biological sample transfer
			Medical Research Council: Guidelines on Ethics in Medical Research: General Principles (2002)
Tanzania	National Health Research Ethics Committee (NHREC) (2002)	Application directly to the NHREC	Ministry of Health (MOH)
			National Institute for Medical Research (NIMR), National Health Research Ethics Committee (NHREC)
			NIMR responsible for coordination of Health Research in Tanzania and coordination of Formation of Institutional Health Research Committees to Formally Approve for Local Health Research
			National Institute for Medical Research, Act of Parliament No. 23, of 1979
			Tanzania Commission for Science and Technology, Act No. 7 of 1986 (COSTECH)
			COSTECH Guidelines on Research Permits and Clearance (2006)
			DNA Act (Act No. 8/09) (2009) Provides guidelines and oversight for laboratories that work with DNA

^a^The following workshop participants provided information from their countries: Burkina Faso, Dr Bocar Kouyaté (Chairman of the National Ethics Committee, Ministry of Health) and Dr Abdoulaye Ouédraogo (Chairman of the IEC, Centre Muraz); Ghana, Dr Cynthia Bannerman and Mr Kofi Wellington (Ghana Health Service Ethical Review Committee); Kenya, Dr Christine Wassuna (Kenyan Medical Research Institute (KEMRI) Ethics Review Committee); South Africa, Dr Yosuf Veriava (Steve Biko Centre for Biomedical Ethics, University of the Witwatersrand)**;** Tanzania, Dr Thomas Nyambo (Member, National Heath Research Ethical Committee (NHREC), Tanzania) and Dr Geoffrey Somi (Deputy Chair, NHREC).

Some countries require both institutional and national ethics approval (Burkina Faso and Kenya). In Kenya, national approval is provided by accredited ethics review committees which are hosted by several research institutions. In Ghana, institutional review is followed by submission to the Ghana Health Service Ethical Review Board if the research facility is part of the Ghana Health Service. South African researchers only require institutional approval, and in Tanzania, only national approval is required. In Ghana and Kenya, institutional scientific review of the research projects precedes ethical review. These differences likely reflect the size and complexity of the research communities. There was a suggestion that the processes in some of the countries may change in the future.

Most of the ethics committees require a description of the informed consent process to be followed during recruitment, together with how confidentiality and anonymity will be maintained and how data and samples will be stored and distributed. In addition, some countries placed an emphasis on a demonstration of local capacity development, and others were concerned about the clear justification of the amount of sample (e.g. blood) collected and how this would be used. There were differences in the approachability of committees in different countries; some actively encouraged applicants to attend ethics meetings to assist with explaining the study in response to the questions raised by the committee members (e.g. in the case of the Navrongo Institutional Review Board (IRB)). This provided an important educational opportunity for both IRB members and researchers. A challenge in the multilingual communities in Africa is the translation of information sheets, consent forms and questionnaires into local languages. In the case of Francophone countries (e.g. Burkina Faso), the full research protocols need to be translated into French.

In some of the countries, the ethics committees perform a monitoring function (e.g. in Ghana genetic and genomic studies as well as clinical trials are monitored by visits to research sites), but in others, logistical and costing constraints inhibited rigorous monitoring. Some ethics committees routinely charged for ethics review, whereas others would do so only in the case of external applications.

A valuable resource on the laws, regulations and guidelines on the protection of human subjects for research is available from the ‘Compilation of Human Research Standards’ (2012 Edition) (Office for Human Research Protections, U.S. Department of Health and Human Services). (http://www.hhs.gov/ohrp/international/intlcompilation/intlcompilation.html). The 2012 version includes information from 14 African countries, including Kenya, South Africa and Tanzania, but not Burkina Faso and Ghana.

## Breakaway sessions

During the breakaway sessions, participants were encouraged to highlight some of the key ethical issues they may have encountered from previous genomic studies they had been involved with, or had reviewed, and to propose recommendations they may provide to overcome the problems or challenges.

## Community engagement

Identifying a ‘community’ and its leadership can be difficult, especially in urban areas. It is important to engage with local health services and to be mindful that in many African communities, ‘blood’ has a cultural context that needs to be understood and taken into consideration when engaging with research participants. Fundamental to the success of all projects is ‘respect’ for people and recognition of the various levels of literacy. Recognising the hierarchies that exist within communities is important in the process of engagement. Engaging through dialogue at each level is important to establish the ‘rules of engagement’ at the onset of commencement of genomic research. Moreover, it is essential that there is effective communication, with easy to understand information, so that expectations are met by appropriate actions. There was general agreement that when community approval was granted through a process of interaction with stakeholders (e.g. in the villages, in the case of rural studies), this would not supersede individual informed consent, and potential participants could still decline participation. Potential misconceptions should be actively addressed and stigmatisation minimised.

## Informed and broad consent

There was an agreement that care should be taken in explaining the nature of the research and potential risks in such a way that they can be understood across different educational levels and cultures. Informed consent would need to be specifically tailored to communities to take this into consideration and information should be translated into local languages as necessary. The concept on broad consent was not familiar to all the participants at the workshop. When seeking ‘broad’ informed consent, participants are required to consent to the use of biological samples and associated data collected for the specified research by the researchers as well as giving their consent for the sharing of samples and data across national borders for future research. In most instances, the research associated with the latter will be unknown to the research team collecting the samples and having the contact with participants. However, the researchers will communicate to participants that future use of their samples and data will be overseen by review processes set up by the H3Africa Consortium. Participants will have an opportunity to ask questions to clarify misunderstandings and will be advised that they have the opportunity to withdraw should they wish to.

There were clear concerns about requesting consent to perform studies that could not be stipulated in the information sheet and also for the wide sharing of data and samples. Some workshop participants found this unacceptable and emphasised that although they understood that it would not be feasible to return to individual participants for re-consenting, they would be more comfortable if all requests for the use of samples over and above the original study request were referred back to the ethics committee concerned with providing the initial approval for the study. The question as to whether it would be reasonable, in the case of AWI-Gen, for Wits as central coordinator of the study, to act as custodian for the study was raised. We did not reach consensus on the question of broad consent for the sharing of samples and data and who should be responsible for making the decisions.

The issue of who the study participants should be and how they should be chosen was raised. In the case where there is not a clear clinical focus (e.g. patients with a specific disease), but the target groups consist of randomly chosen individuals from specific geographic areas, the group felt that there should be an attempt at equity in choosing participants (e.g. randomization). Strategies would need to be developed to ensure that there is no discrimination applied.

Questionnaires and questions should be carefully aligned with the objectives of the research study. Creating an expectation of health-related feedback to individuals should be avoided and addressed directly with the participants at the outset of the project. Community feedback and providing a context for the research findings is important. The question of whether participants should be informed of findings that are unrelated to the study, but that may impact their health or that of their children (e.g. sickle cell carrier status), was hotly debated. There was, however, general agreement that when appropriate, participants who require medical care based on phenotypic findings (e.g. high blood pressure) should be referred to the national health system infrastructure.

## Sample and data sharing

A characteristic of genomic research generally, and of our project more specifically, is that it usually involves international collaboration. In addition, funding conditions for AWI-Gen require us to make data and samples available for secondary analysis and use. It was recognised that there is a need to balance access with the interests of the participants, the ethics committees (in their capacity as protectors of participants) and the funders and that sharing needed to be a two-way process.

Many workshop participants had not previously encountered or reviewed research of this nature, and not all the participants felt confident that their countries and committees had developed adequate policy frameworks to guide this kind of research. Participants felt that it would be very important to clarify ownership over resources, most notably regarding samples. A question arose about whether ownership rested with individual participants, research groups or their institutions and countries. The group also questioned whether and how ownership would be transferred in the case of secondary use. When samples are shared, this needs to be done in accordance with the legislative requirements of the different countries. In the experience of the workshop participants, material transfer agreements (MTAs) are considered a good tool to facilitate international sharing of samples whilst protecting the interests of the local institutions. It was felt that there needs to be appropriate recognition of the contribution of local researchers and institutions (including infrastructure and personnel costs) when samples are shared. Authorship and acknowledgement in publications is therefore important, and guidelines need to be developed and outcomes documented.

During the discussions, several of the ethics committee members emphasised that they would require ethics review committee approval or permission for secondary use of samples for projects not stipulated in the original applications. This raises a potential conflict (or logistical challenge) within the H3Africa Consortium, where the intention is to have centralised consortium-specific data and sample access processes where individual project leaders may not be directly represented. Further exploration toward a solution will require wider debate.

Although there was consensus that it is necessary to share samples for large international research studies across national borders, there were country-specific stipulations. In Kenya, clear justification needs to be made for why research cannot be done in Kenya before permission is granted to export samples out of the country. In South Africa, an export permit is required from the National Department of Health. Material transfer agreements and memoranda of understanding were considered important tools to ensure appropriate regulation with regard to sample and data sharing.

## Benefit sharing

The question of how genomic research can benefit the people and populations it involves is pertinent, particularly in the African continent where many communities continue to live in poverty. However, it is not straightforward to determine exactly what would constitute ‘benefit’ and how and who this should be shared with. The group identified a number of stakeholders that ought to be considered, including research participants, their communities, the broader patient group or society and the research community (local, national and international). For the AWI-Gen project, and possibly for H3Africa research more widely, the focus on African leadership and capacity development could constitute the primary form of non-scientific benefit out of this research. In addition, it was suggested that recruitment could be accompanied by information about the health implications of obesity. This is particularly relevant in the case of AWI-Gen, which focuses on a growing public health problem in Africa. Knowledge about the dangers of excess body fat is still limited, and meeting participants felt that AWI-Gen could provide some additional benefit through public education. Similarly, a project like AWI-Gen could link to existing patient organisations to provide relevant information. A last important issue of concern - often reflected in ethics discussions in Africa - relates to the use of genomic samples and data to generate commercially valuable products. Meeting participants generally felt that if this was to happen, then research participants or their communities should have an opportunity to benefit from or share in such financial gain.

## Summary

Robust discussion and debate suggested that more discussion is necessary across participant countries to ensure a common understanding of genomic research and wide sharing of samples and data. Such discussions need to be premised on a thorough knowledge of genomics and global trends toward open and wide access. In many African countries, national guidelines and regulations for research are relatively broad, without providing insight into genomic research, and the individual ethics committees make independent decisions. Among participant countries, some ethics committees (Kenya and South Africa) have considerable experience in reviewing genomic projects, but in others, little expertise currently exists. All, however, indicated a need to further strengthen capacity for reviewing genomic projects in line with national and international guidelines for good practice and to ensure maximum benefit to participant communities.

The H3Africa Consortium is providing a platform to discuss and debate the issues that have been raised and is developing policies and guidelines that will be important in a much wider context for genomic research in developing countries. Its prime objective is to promote genomic research capability on the African continent for the benefit of the health of its people and the global community.

## Competing interests

The authors declare that they have no competing interests.

## Authors' contributions

MR and OS planned and convened the workshop. MR, JdV, HS, and SN facilitated the breakaway sessions and contributed to the writing of the paper. All authors read and approved the final manuscript.

